# Sentiment Analysis for Words and Fiction Characters From the Perspective of Computational (Neuro-)Poetics

**DOI:** 10.3389/frobt.2019.00053

**Published:** 2019-07-17

**Authors:** Arthur M. Jacobs

**Affiliations:** ^1^Department of Experimental and Neurocognitive Psychology, Freie Universität Berlin, Berlin, Germany; ^2^Center for Cognitive Neuroscience Berlin, Berlin, Germany

**Keywords:** sentiment analysis, computational poetics, emotional figure profile, hybrid hero potential, machine learning, digital humanities, neuroaesthetics, literary reading

## Abstract

Two computational studies provide different sentiment analyses for text segments (e.g., “fearful” passages) and figures (e.g., “Voldemort”) from the Harry Potter books (Rowling, 1997, 1998, 1999, 2000, 2003, 2005, 2007) based on a novel simple tool called *SentiArt*. The tool uses vector space models together with theory-guided, empirically validated label lists to compute the valence of each word in a text by locating its position in a 2d emotion potential space spanned by the words of the vector space model. After testing the tool's accuracy with empirical data from a neurocognitive poetics study, it was applied to compute emotional figure and personality profiles (inspired by the so-called “big five” personality theory) for main characters from the book series. The results of comparative analyses using different machine-learning classifiers (e.g., AdaBoost, Neural Net) show that *SentiArt* performs very well in predicting the emotion potential of text passages. It also produces plausible predictions regarding the emotional and personality profile of fiction characters which are correctly identified on the basis of eight character features, and it achieves a good cross-validation accuracy in classifying 100 figures into “good” vs. “bad” ones. The results are discussed with regard to potential applications of *SentiArt* in digital literary, applied reading and neurocognitive poetics studies such as the quantification of the hybrid hero potential of figures.

*I tried to gain an idea of the number of the more conspicuous aspects of the character by counting in an appropriate dictionary the words used to express them…I examined many pages of its index here and there as samples of the whole, and estimated that it contained fully one thousand words expressive of character, each of which has a separate shade of meaning, while each shares a large part of its meaning with some of the rest*.

— Francis Galton, Measurement of Character, 1884

## Introduction

Computational analysis and modeling of narratives or poetry still present a wealth of challenges for research in digital literary studies, computational linguistics, machine learning, or neurocognitive poetics (e.g., Nalisnick and Baird, 2013; Ganascia, 2015; Jacobs, 2015a, 2018b). A key issue concerns the extent to which computers can evaluate the emotional information encoded in spoken or written texts, i.e., what is typically called sentiment analysis (SA). While there is considerable progress in SA in the last 20 years (e.g., Liu, 2015), when it comes to poetic texts such as Shakespeare sonnets (Simonton, 1989; Jacobs et al., 2017) new challenges like the prediction of *aesthetic emotions* via SA tools must be tackled. First attempts at quantifying e.g., the beauty of words (Jacobs, 2017), the most beautiful lines of poetry (Jacobs, 2018a,b) or the “aptness” of poetic metaphors (Jacobs and Kinder, 2017, 2018) are encouraging, but the lack of both specialized SA tools and empirical data allowing to assess their descriptive accuracy and predictive validity slows down progress.

A special aspect of SA addressed in this paper concerns the computational modeling of the emotional facets of a given figure or character described in natural language text (e.g., Egloff et al., 2016) or the emotional relationships between characters e.g., via character-based kernels (Elsner, 2012) or character-to-character *SA* (Nalisnick and Baird, 2013). Here I'd like to propose a simple heuristic tool for computing *Emotional Figure Profiles* and *Figure Personality Profiles* for characters in stories which can be used, for example, as a means to quantify the “hybrid hero potential” of figures in novels, an extension of the digital modeling of figure complexity (e.g., Klinger, 2018). In order to tackle this issue I use a vector space model (VSM)-based SA tool that has proven useful for computing the emotion potential of poems and of excerpts from Rowling's (1997, 1998, 1999, 2000, 2003, 2005, 2007) *Harry Potter* book series (Jacobs, 2018a,b).

## Three Approaches to SA

The wealth of SA methods can be categorized into three broad classes: dictionary or word list-based, VSM-based, and hybrid methods (Taboada et al., 2011; Jacobs, 2018b). The first method determines the positivity/negativity value (i.e., its valence) of a word from the test text by looking it up in a reference word list or “prior-polarity lexicon” that contains the information, typically based on human rating data (e.g., Whissell et al., 1986; Bestgen, 1994; Wiebe et al., 2005). Following Stone et al.'s (1966) early content analysis tool, this method uses word lists with rating data like the *Berlin Affective Word List* (BAWL; Võ et al., 2006, 2009; Briesemeister et al., 2011; Jacobs et al., 2015), the *Affective Norms for German Sentiment Terms* (ANGST; Schmidtke et al., 2014), or the norms by Warriner et al. (2013). If one adheres to the theory that valence is a semantic superfeature that results from a yet unknown integration of both *experiential* and *distributional* data at least partially represented in associative activation patterns of semantic networks (Andrews et al., 2009; Jacobs et al., 2016a), then SA based on human ratings is the closest to the experiential aspect one can get. Theoretically, the valence value of a given word would thus be computed in the brain from (1) neural activation patterns distributed over the sensory-motor representations of the word's referents (*experiential* or embodied aspect) and (2) information about the linguistic company the word keeps (e.g., Harris, 1951), as estimated by the size and density of its learned context (*distributional* aspect). However, optimally such databases cover each (content) word in the test text, although, in reality, a limited hit rate of the database is often the biggest problem of this method, especially when dealing with highly literary or ancient text materials (cf. Jacobs and Kinder, 2017; Jacobs, 2018b). A related more general problem is the language, if there are no or only limited word lists in the language of a researcher's country. Simply translating existing English lists into another language without empirical cross-validation has its own problems (Schmidtke et al., 2014) and sensitivity to emotional content varies across languages which differ considerably in their emotion vocabularies (e.g., Conrad et al., 2011; Veltkamp et al., 2012; Hsu et al., 2015b). Since running comparative studies in several languages—as in the afore-cited studies from my group—is an important aim of the Neurocognitive Poetics perspective (Jacobs, 2011, 2015a,b,c, 2016, 2017; Willems and Jacobs, 2016; Nicklas and Jacobs, 2017; Jacobs and Willems, 2018) a method is required that works for many languages. Moreover, collecting human rating data is costful and there are methodological and epistemological issues about the reliability and validity of ratings, especially when they are turned from a dependent variable (i.e., a “subjective” behavioral measure in response to a stimulus) into an independent variable (i.e., an “objective” predictor of say the positivity of a text; cf. Westbury, 2016).

The second method introduced by Turney (2001; cf. also Turney and Littman, 2003) offers a computational alternative which requires no access to human rating data and can work in about any language for which training corpora and semantic vectors are available or can be created (e.g., fasttext https://fasttext.cc/docs/en/pretrained-vectors.html). It uses an unsupervised learning approach to SA based on VSMs and a label list which can be thought of as prototypes or more or less ideal examples of positive and negative *semantic orientation* or *valence* (for example, GOOD, NICE vs. BAD, NASTY). This method thus concerns the *distributional* aspect of valence and works by computing the semantic relatedness between the words of the test text and the labels using knowledge-based, dictionary models, vector space or neural net models (e.g., wordnet; Miller, 1995; latent semantic analysis/LSA; Deerwester et al., 1990 or word2vec/w2v; Mikolov et al., 2013). The labels must be part of the training corpus used to generate the similarity vectors (e.g., via LSA, wordnet or w2v). If a given test word is—on average—more similar to a set of positive labels like GOOD than to the opposite set, it will be classified as having a positive valence and vice versa. Naturally, parameters like the size, representativeness or specificity of the training corpus, the validity of the vector space or other models (incl. the similarity metric) used to compute semantic similarities present challenges for this 2nd method 2. Perhaps the biggest of those concerns the quantity, quality, and context-sensitivity of the labels whose choice can be subjective, intuitive or heuristic (Turney and Littman, 2003; e.g., Jacobs, 2017), or theory-guided (e.g., Westbury et al., 2015). Turney and Littman (2003) argued that their unsupervised tool with a training set of only 14 labels has the advantage of not requiring re-training for each new domain unlike supervised SA tools.

The third method is a hybrid between the first two. Like the 2nd, it starts with estimating—using some training corpus—the similarity between the test text words and a list of labels for which valence rating data must be available. It then computes the valence value for a test word as the average of the ratings of its *k nearest neighbors* in the vector space (Taboada et al., 2011; Bestgen and Vincze, 2012; Recchia and Louwerse, 2015). Thus, Method 3 combines the advantages as well as the disadvantages of the two former methods.

The pros and cons of these different methods have been discussed in detail elsewhere (Mandera et al., 2015; Westbury et al., 2015; Hollis et al., 2017). Suffice it to say that if rating data are not available or fail to cover a reliable percentage of the words in the test text (cf. Jacobs and Kinder, 2017), then the second method is the only viable one. In this paper I would like to test the validity of a novel variant of this method (called *SentiArt*) for doing SA of literary texts and characters within the emerging field of Neurocognitive Poetics.

Word list-based methods can only be used for the present task of determining the valence of characters from narratives (see Study 2), if they contain the proper names of the characters in the story, e.g., “Voldemort.” For the affective word lists in German developed in my group (i.e., the BAWL or ANGST), this is not the case. However, VSM-based techniques can be applied to the extent that the semantic vectors are computed from an adequate training corpus, e.g., in the present case the entire Harry Potter book series (Rowling, 1997, 1998, 1999, 2000, 2003, 2005, 2007) in its German translation (~1.4 million tokens, ~40.000 types) which naturally contains the names of the protagonists and other figures.

## Present Study

The present two computational poetics studies are part of the larger above-mentioned Neurocognitive Poetics perspective and aim at proposing and testing a simple, easy-to-use tool for computing emotional figure, and figure personality profiles for characters in literary texts such as stories, novels, plays or ballads. Study 1 aims at testing the feasibility of *SentiArt* as a simple VSM-based tool for computational poetics studies in multiple languages. Having obtained encouraging results from study 1, study 2 introduces the computation of emotional figure profiles and figure personality profiles for characters from the Harry Potter book series.

### Study 1. Classifying Text Segments From Harry Potter as “Joyful,” “Fearful,” or “Neutral”

In a 1st computational study I used a comparative predictive modeling procedure—successfully applied in previous research (Jacobs et al., 2016a, 2017; Jacobs, 2017; Jacobs and Kinder, 2017, 2018)—to test how well the sentiment labeling data of a *test set* could be predicted by the results of those of a *training set* used to train different classifiers. Overall 120 text segments from Harry Potter that were labeled as “happy,” “fearful,” or “neutral” in a previous empirical study (Hsu et al., 2015a,b) were used as stimulus material. Five classifiers—as implemented in the Python toolbox *Orange* (Demsar et al., 2013)—were used for predictive modeling: Adaboost (AB, with Simple Decision Tree), kNearestNeighbors (kNN), Logistic Regression (LR), Naïve Bayes (NB), and Neural Network (MultiLayerPerceptron/MLP). The task for the classifiers was to predict the sentiment category of the segments based on the input provided by different computational SA tools (SATs).

#### Computing the Text Emotion Potential

An early empirical study by Bestgen (1994) showed that the “affective tones” of sentences and entire texts can well be predicted by lexical valence as determined by a word-list based method. More recent neurocognitive studies confirming this idea showed the power of text valence for evoking emotional reader responses as measured by their underlying neuronal correlates (Altmann et al., 2012, 2014; Hsu et al., 2014, 2015a,b,c).

To get an idea about *SentiArt's* relative performance, it was compared with those of two other well-established SATs, both implemented in publically available software packages such as *Orange* or *NLTK* (Bird et al., 2009). The 1st, *VADER* (Hutto and Gilbert, 2014), is a popular word list- and rule-based procedure which computes a continuous score for each text (ranging from negative to neutral to positive values) and appends a total sentiment score called compound^1^. It can deal with some forms of negation or punctuation emphasis in texts. Like VADER, the 2nd SAT (*HU-LIU*; Hu and Liu, 2004) also belongs to the first class of SA methods and computes a single normalized sentiment score for a text (ranging from negative to neutral to positive). These scores were obtained directly from *Orange* for each of the 120 text segments. The 3rd SAT, belonging to the second method class (*SentiArt)*, is derived from the theory-guided *computational semantics* perspective allowing to compute theoretical values successfully predicting human ratings for imageability (Westbury et al., 2013), valence and arousal (Westbury et al., 2015) or lexical aesthetic potential—for a variety of materials such as single words, lines from Shakespeare sonnets or literary metaphors (Jacobs, 2017, 2018a,b; Jacobs and Kinder, 2018). In this perspective, valence, for example, is computed as a semantic association compound based on the relatedness of a target word with each of N labels in the positive and negative lists. Westbury et al. (2015) tested 12 models based on psychological emotion theories and established the “Ekman99” (Ekman, 1999) model with 12 labels (seven positive labels, such as HAPPINESS or PRIDE, and five negative ones such as DISGUST or FEAR), as the best: it accounted for about 34% variance in the validation set of > 10.000 human valence ratings from Warriner et al. (2013) (For details see Table 2 in Westbury et al., 2015).

#### Method

**Figure 1:**
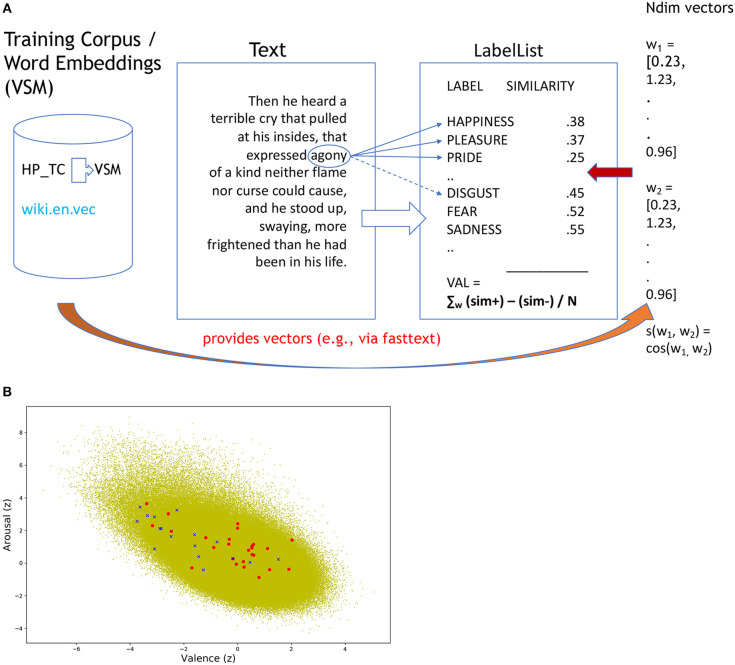
**(A)** Schematic illustrating the procedure for computing the Emotion Potential Space shown in **(B)**. **(B)** Emotion Potential Space with Distribution of valence (x-axis) and arousal (y-axis) values for the >2 million words from the wiki.en.vec corpus (“green cloud”) and those from two sample segments of the Harry Potter stimuli (“fear” segment = blue crosses; “happy” segment = red circles, both increased in size for better visibility).

To establish e.g., the valence of a text segment with SentiArt, the procedure was straightforward and easy to replicate by researchers not necessarily trained in NLP methods^2^. In a 1st step, an appropriate—general or task-specific—training corpus such as the above-mentioned “Harry Potter” corpus (HP_TC in Figure 1A) is created by merging all texts (e.g., seven books) into a single compound and the corresponding VSM is computed, e.g., by running the easy-to-use fasttext tool (https://fasttext.cc/docs/en/pretrained-vectors.html). If a specific training corpus is not required, one can use the procedure described on the *fasttext* homepage (https://fasttext.cc/docs/en/english-vectors.html) to directly download the VSM providing e.g., 300d vectors (Ndim vectors on the right side of Figure 1A) for each of >2 million words (e.g., in the original, uncleaned version of *wiki.en.vec*). Now all is set up for the 2nd step, i.e., the computation of the semantic relatedness values between each word in the VSM and the labels in the label list. For example, computing the cosine similarities between the target word AGONY and the first three positive and negative labels of the *Ekman99 model* set of labels (HAPPINESS, PLEASURE, PRIDE) empirically validated in previous studies with both English and German text materials (Westbury et al., 2015; Jacobs, 2018a,b) would yield a mean value of 0.33 for the positive labels and of 0.51 for the negative ones. Subtracting one from the other gives a valence of −0.18 thus suggesting that the word AGONY has a negative valence. This procedure was applied to all words in the VSM (*wiki.en.vec*), computing both valence and arousal values.

The procedure establishes a 2D Emotion Potential Space (valence X arousal) with > 2 million entries (available as an.xlsx table from the author), illustrated in Figure 1B, which could serve as a reference space for many future SA studies. Thus, each word of a given test text (e.g., a segment from Harry Potter) can easily be located within this space (e.g., using the.xlsx table) thus receiving a standardized (relative) valence and arousal value. In a 3rd step, the average scores for the text segments of interest (e.g., those rated as fearful or joyful by human readers) are computed so they can serve as input (predictors) for the classifiers.

#### Predictive Modeling

After computing the three SAT features (VADER's compound, HU-LIU's sentiment, and SentiArt's valence) for each of the 120 text segments, the features were standardized and used as input for five classifiers implemented in *Orange* to check the performance accuracy of the three SATs in predicting the sentiment category of a test set (after being trained on a training set of 70% of the 120 segments). The random sampling was stratified (i.e., balanced across the three text categories) and repeated 100 times with varying training and test sets to obtain stable results. As a control condition, I used LSA (Deerwester et al., 1990), also implemented in *Orange*—and which is not a SAT as such–, to check how well it classified the text segments without using special sentiment features.

Please note that the present study was not designed as a “benchmark” test for SATs. The SATs are not directly comparable, since they belong to different method classes. Thus, both VADER and HU-LIU compute a univariate sentiment feature—theoretically reflecting the “experiential” aspect of valence—which is based on a list of previously rated, special “sentiment” words (Vader: ~7.500 entries; https://www.cs.uic.edu/~liub/FBS/sentiment-analysis.html#lexicon).; (Hu-Liu: ~6.800; https://www.cs.uic.edu/~liub/FBS/sentiment-analysis.html#lexicon). In contrast, SentiArt is a multivariate SAT usually computing four lexical features (valence, arousal, emotion potential, aesthetic potential) and two interlexical ones (valence span, arousal span) to predict the multiple “distributional” aspects of a text's sentiment (incl. function words which can play important roles in poetry processing by altering the aesthetic potential of e.g., entire lines; Jacobs, 2018b). Rather than in “benchmarking” I was interested in testing the feasibility of SentiArt as a simple unsupervised learning SAT for neurocognitive poetics studies in multiple languages and needed some reference tools (working in English) to be able to better interpret SentiArt's results for the present English Harry Potter texts. Since both Vader and Hu-Liu were implemented in *Orange* I chose them for practical reasons.

#### Results and Interim Discussion

**Figure 2:**
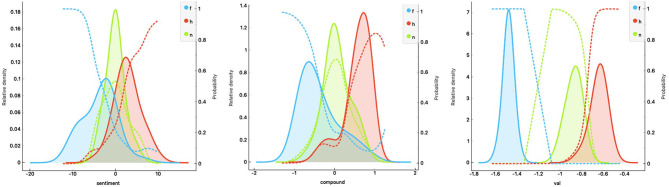
Distributions (with probabilities = dotted lines) of the feature values across the three text categories (f = fear, h = happy, n = neutral; left panel: HU-LIU, middle: VADER, right: SentiArt; val = valence).

The results summarized in Table 1 show the classification scores^3^ for each of the three SATs and the LSA. The present—purely descriptive—classifier comparison shows an optimal performance for SentiArt's *valence* feature (with Logistic Regression) and smaller scores for VADER's *compound* feature (with Neural Net) and HU-LIU's *sentiment* feature (with Logistic Regression). The performance of the control method (LSA), though inferior to the others, suggests that the abstract semantic features computed by LSA still capture affective aspects that allow to classify (to a certain extent) texts into sentiment categories. A look at Figure 2 shows that SentiArt's valence feature splits the three categories better than the other two. Given that SentiArt computes a feature value for each word in the text this could be expected.

**Table 1:** F1 scores for the three SATs obtained with five classifiers (stratified random sampling, 70/30, average values for 100 repetitions).

**Classifier/Method**	**Hu-Liu**	**Vader**	**SentiArt**	**LSA**
Neural network	0.693	0.742	0.915	0.620
Logistic regression	0.700	0.716	1.000	0.660
AdaBoost	0.613	0.674	0.972	0.420
kNN	0.626	0.675	0.972	0.344
Naive bayes	0.676	0.713	0.838	0.637

It should be emphasized that the present results do not show that either VADER or HU-LIU are generally less well-performing than SentiArt. In contrast to SentiArt, they both are general, widely applicable SATs well-validated in e.g., many SAs of social media texts (Hutto and Gilbert, 2014; Liu, 2015). Also, revised, more sophisticated versions of at least the HU-LIU SAT exist (Liu, 2015) which may yield different results, but are not (yet) implemented in *Orange* and could thus not be used here. The point is that within the confines of the present special materials tested in several neurocognitive poetics studies (Hsu et al., 2015a,b,c), SentiArt's performance can be considered as competitive.

### Study 2. Computing the Emotional and Figure Personality Profiles for Main Characters in “Harry Potter”

As far as I can tell, so far VSM-based SATs have not been used to estimate the valence or emotion potential of characters in stories, but it seems to be a natural application which is of special interest for digital literary and neurocognitive poetics studies, e.g., for predicting identification and empathy, both important factors driving immersive responses (e.g., Jacobs and Lüdtke, 2017). There is related work, however. Thus, for example, Elsner (2012) used a word-list based method to compute the “emotion” of characters such as “Miss Elizabeth Bennet” from Jane Austen's *Pride and Prejudice* by counting all emotional words in paragraphs that featured only one character and adding them to the character's total. Also using a word-list based method, Nalisnick and Baird (2013) mined for character-to-character sentiment in Shakespeare's *Hamlet* by summing the valence values over each instance of continuous speech working on the simplifying assumption that sentiment was directed toward the character that spoke immediately before the current speaker. As already mentioned, Egloff et al. (2016) used IBM Watson to compute Hamlet's or Othello's “big 5.” More recently, Klinger (2018) presents a VSM-based approach for computing the complexity of figures in stories (e.g., Eschenbach's “Parcival”) using lexical diversity and information content measures. Figure complexity is an interesting feature related to what I'd like to call the *hybrid hero potential*: from Homer's *Iliad* to Gilligan's *Breaking Bad* fiction protagonists have been depicted with conflicting features or traits to make them more interesting/attractive to readers, listeners or viewers. The emotional figure profile and figure personality profile introduced here can help quantify this hybrid hero potential to predict aesthetic responses (“liking,” “interest”) of readers, for example.

#### Emotional Figure Profile

The simple idea behind computing an emotional figure profile is that the strength of semantic associations between a character (name) and the prototypical “emotion words” contained in the label list gives us an estimate of their emotion profile. Thus, the figure-based context vectors underlying the emotional figure profile specify the *affective context profile* of a figure relative to other figures in the story. They are merely suggestive and do not directly specify emotional or social “traits” of a figure, for example via recognizing adjectives or phrases directly referring to the figure (e.g., “X is a dangerous person”) as in aspect-based SA (Liu, 2015).

Most SATs are univariate, i.e., they compute a single value, e.g., *HU-LIU*. Based on previous work mainly using a word list-based approach (Jacobs et al., 2016a,b), I recently proposed the computation of the *emotion potential* of words, sentences or chapters as an extension of univariate SA (Jacobs, 2015a). In contrast to the latter which attributes only a valence value to each unit of analysis, the emotion potential combines two of the three dimensions in Wundt's (1874) classical psychological theory of emotion, i.e., valence and arousal. Thus, the emotion potential/EP of a word is computed as: EP_w_ = |valence_w_| ^*^ arousal_w_, and estimates the bivariate potential with which a word or larger text unit can elicit emotional responses in readers. In psychology, after more than 150 years there still is no consensus which of the two “big” emotion theories is correct (cf. Schrott and Jacobs, 2011): “dimensional” theories of emotion (e.g., Wundt's valence and arousal) or “discrete” ones (e.g., Ekman, 1999). The VSM-based variant of the emotion potential therefore takes both theoretical approaches into account since its computation is based on discrete labels (e.g., *joy, fear* etc.; see Appendix B in the Supplementary Material in Jacobs, 2018a), but its output is a continuous value on the bipolar “negativity-positivity” and “calming/arousing” dimensions, respectively.

To compute the emotional figure and figure personality profiles for characters from the Harry Potter book series I proceeded much like in Study 1. I first generated a task-specific training corpus merging the texts from all seven Harry Potter books (in their German translation from the “childlex” corpus; Schroeder et al., 2015) and then computed the corresponding VSM using the fasttext tool (https://fasttext.cc/docs/en/pretrained-vectors.html) with the following parameter specifications in addition to the default values: skipgram model with 300 dimensions, no character n-grams, minimal count = 0, deterministic thread (so one can replicate the vectors). The resulting *HPde.vec* VSM used ~85 k 300d vectors extracted from ~58 k sentences and, thus, these vectors are not representative for or generalizable to other training corpora: using different VSMs will lead to different results. Still, this VSM appears to be the most adequate task-specific one for the present purposes.

**Figure 3:**
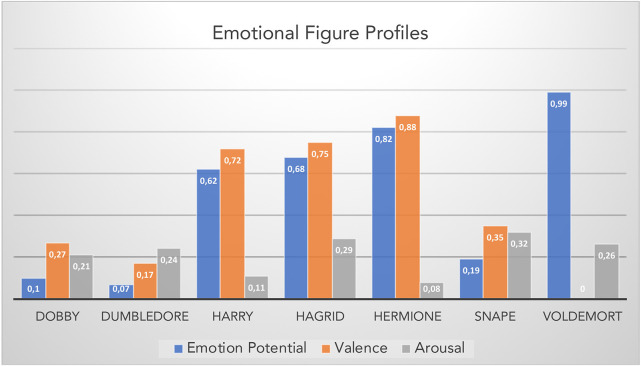
Emotional figure profiles for seven main characters representing percentiles of their raw valence, arousal, and emotion potential scores within the Harry Potter corpus based on a sample of 100 figures (see Appendix in the Supplementary Material).

Like in Study 1 I then “sentiarted” each of the words in the “Harry Potter” corpus and located the names of seven main characters from Harry Potter in the resulting 2d space. The raw scores (Arousal, Valence, Emotion Potential) were transformed into percentiles based on a sample of 100 figures appearing in the book series (from “Albus” to “Wilkes”; see Appendix in the Supplementary Material). Figure 3 shows the Emotional Figure Profiles for these seven main characters.

The example emotional figure profiles in Figure 3 would suggest “Harry,” “Hermione,” and “Hagrid” as the protagonists of the stories with the highest relative valence values. Expectedly, “Voldemort” has the lowest valence percentile (0), but a very high emotion potential on account of his relatively high arousal and very high negative valence raw score (−0.03). Lacking empirical data (e.g., reader ratings) that could validate these estimations I refrain from any interpretation beyond face validity considerations. Suffice it to say that the present emotional figure profiles appear solid enough to serve as predictors for empirical studies of reading (e.g., Jacobs, 2015b). Thus, one could have participants read a variety of excerpts from the Harry Potter series and collect ratings (for liking, familiarity etc.) for a set of main figures, similarly to ratings regarding the emotion potential of the entire excerpt (e.g., Hsu et al., 2015a). A particularly interesting prediction concerns those figures whose arousal and valence values are discrepant, e.g., “Hagrid” or “Voldemort”: the 1st has a low arousal and high valence whereas the 2nd has the opposite profile. Together with the figure personality profiles discussed in the next section, such discrepancies might contribute to the hybrid hero potential. Peripheral-physiological measures such as heart rate variability or electrodermal activity could be sensitive to such discrepancies (Jacobs et al., 2016b) as could be brain activity measures that are sensitive to mental conflicts (e.g., Hofmann et al., 2008).

#### Figure Personality Profile

In this section I present some more differentiated computational “personality profiles” that are inspired by research in personality and clinical psychology, in particular so-called lexical approaches to personality assessment. These are based on common language descriptors and therefore on the association between words rather than on neuropsychological experiments. More specifically, the popular *OCEAN* model (perhaps better known as “big5”; Norman, 1963) assumes that five global factors (Openness to experience/Intellect, Conscientiousness, Extraversion, Agreeableness, and Neuroticism/Emotional Instability) capture personality characteristics that are most important in people's lives. These are hypothesized to eventually become part of their language and are more likely to be encoded into language as a single word than others. The above mentioned work by Egloff et al. (2016) already applied the big5 model “to help understand character in Shakespeare” and create personality profiles for main characters in his plays such as “Hamlet.” The authors used IBM Watson (Ferrucci, 2012) for their analyses.

Here, I used the same simple technology as for the multivariate SA and computation of the emotional figure profile shown in Figures 1, 3, i.e., a combination of a VSM and task-specific label lists. The labels were chosen on the basis of extensive pilot studies examining candidate items for each of the big5 dimensions and their underlying more specific primary factors loosely inspired by Osgood et al.'s (1957) semantic differential, Goldberg's (1992) 100 unipolar markers and transparent bipolar inventory, as well as Thompson's (2008) “International English Big-Five Mini-Markers.” Naturally, only labels contained in the German Harry Potter VSM's vocabulary could be used. The procedure started with a representative seed word [e.g., CURIOUS for the positive pole of the Openness dimension; cf. “the defining method” of Turney and Littman (2003)] and then proceeded with searching for synonyms and antonyms of this seed word using the semantic relatedness values of the VSM. The seed words for the 10 poles of the five dimensions were: Openness/Intellect (unintelligent-curious), Conscientiousness(risky-staid), Extraversion(timid-energetic), Agreeableness(distrustful-friendly), and Neuroticism (calm-nervous).

It should be noted that the present approach termed “pseudo-big5” is only loosely inspired by the OCEAN model and that this computational lexico-semantic approach bears only partial similarities to key words appearing in the original or revised “big5” questionnaires (e.g., Costa and McCrae, 1988). Naturally, I make no claims regarding the validity of this “pseudo-big5” approach as a scientific tool for assessing personality profiles of real persons.

#### Pseudo-Big 5

**Figure 4:**
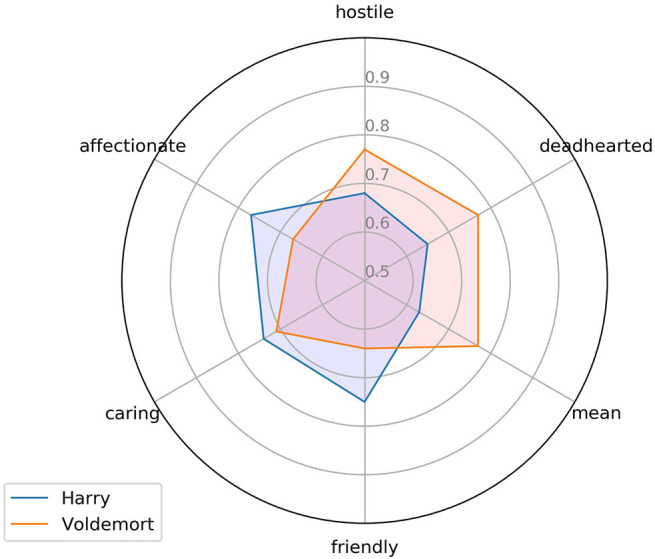
Scores on six representative labels for the “Agreeableness” dimension for two main characters from *Harry Potter* (translated from the German originals by the author). The labels “affectionate,” “caring,” and “friendly” hypothetically represent the positive pole of the dimension, the other three the negative one^6^.

**Figure 5:**
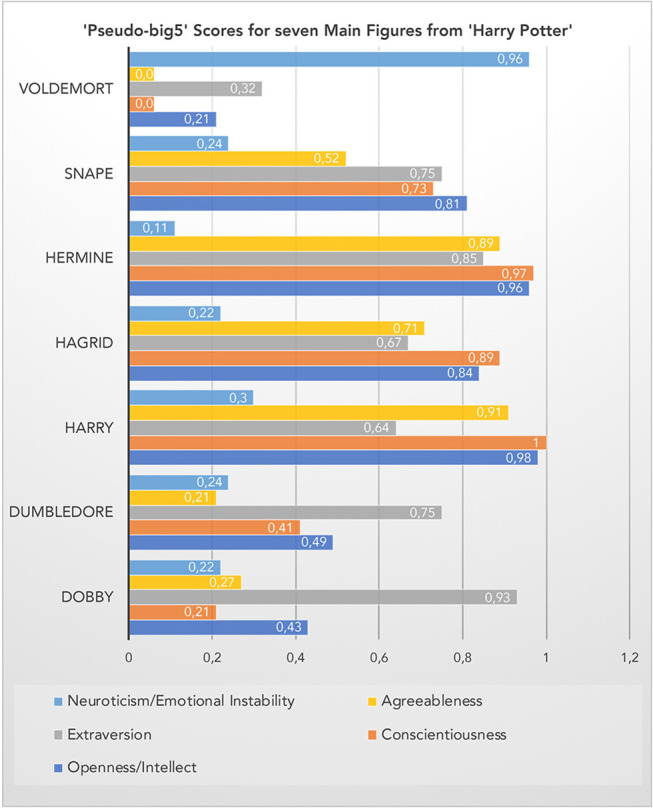
Pseudo-big5 scores for seven main figures. The scores are percentiles based on a sample of 100 figures appearing in the book series (see Appendix in the Supplementary Material).

The exemplary data in Figure 4—based on the HPde.vec model—show the semantic relatedness scores computed for the words “Harry” and “Voldemort” with six labels hypothetically representing the negative and positive poles of the Agreeableness dimension of the pseudo-big5 model (cf. Appendix A in the Supplementary Material). To the extent that the present pseudo-big5 model is of any heuristic value, the data in Figure 4 suggest that the “Harry” character is more closely related to the semantic concepts of “affectionate,” “caring,” and “friendly” standing for the positive pole of the dimension, while “Voldemort” is more related than “Harry” to the negative concepts of “deadhearted,” “hostile,” and “mean.” Still, the data also show the inherent ambiguity/complexity (“hybrid hero potential”) resulting from this method: thus, Voldemort, although more deadhearted than Harry, also scores > 0 on the 'affectionate' dimension, while Harry, although being more friendly than Voldemort, also scores a.68 value for “hostile.” The overall VSM-based raw scores for “Harry” vs. “Voldemort” on the Agreeableness dimension were: Harry 0.063, Voldemort −0.07, i.e., the “Harry” figure would be considered as overall more “agreeable” than “Voldemort,” if these scores were on an interval scale. However, as for the data in Figure 3. I assumed that this is not the case and rather used percentiles to estimate each figure's score, based on the pseudo-big5 scores for 100 characters from the book series. The same procedure was applied for the other four dimensions of the pseudo-big5 model. The results for seven main characters are shown in Figure 5.

Within this selective set of seven characters, the top scorer on the Openness (to experience), Conscientiousness and Agreeableness dimensions is “Harry,” while “Voldemort” takes the lead on the Neuroticism dimension. Interestingly, “Dobby” is the winner on the Extraversion dimension. In the absence of empirical data, I leave it up to readers of this article to judge the face validity of these tentative results. Their heuristic value is clear, though, and can readily be tested e.g., by an experiment with human readers who are invited to judge these seven (or more) characters on scales borrowed from the “big5” personality inventory. A quantification of the hybrid hero potential in future empirical studies investigating its influence on (aesthetic) emotional reader responses could make use of data as those in Figures 4, 5 by using, for instance, only opposite categories such as risky-cautious, good-bad or nice-nasty and computing corresponding ratios.

#### Figure Identification

**Figure 6:**
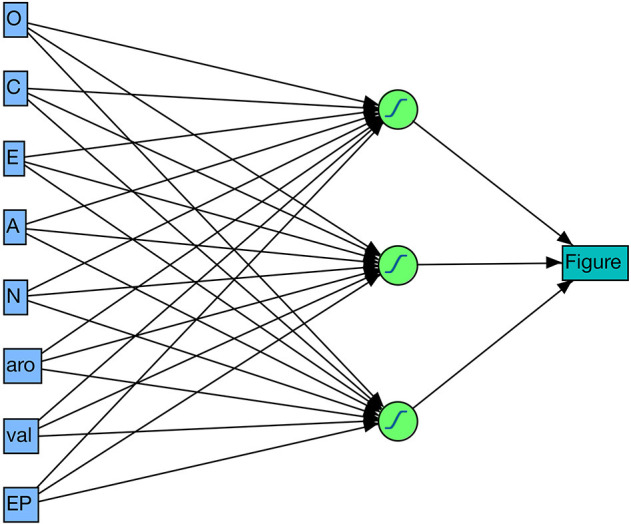
Neural net architecture with eight input, three hidden, and one output unit(s).

As a first test of the usefulness of the computational pseudo-big5 I checked how well the 100 figures (see Appendix A in the Supplementary Material) could be identified on the basis of the three features from the emotional figure profile (valence, arousal, emotion potential) and the five big5 features from the figure personality profile (i.e., O-C-E-A-N). For this I used the simple “Neuronal model” from the JMP14 Pro statistics software (SAS Institute Inc., Cary, NC, 1989–2007) shown in Figure 6 since it allows an estimation of the feature importances not only for the total sample of 100 characters, but also for each individual one^4^. The model fit was excellent (entropy *R*^2^ = 0.999^5^, misclassification rate = 0) with the overall most important features—according to the total effect, dependent resampled inputs option—being: emotion potential/EP = 0.63, N = 0.61, A = 0.6, E = 0.54, aro = 0.53, O = 0.38, C = 0.36, val = 0.12). Thus, for perfectly identifying each of the 100 figures the neural net mainly used information about the emotion potential, neuroticism, and agreeableness scores, mixing in data about the extraversion and arousal values, and—to a lesser extent—the openness, conscientiousness and agreeableness scores. Looking at the individual feature importances for the seven main figures in Table 2, it can be seen that the neural net model flexibly uses the entire spectrum of (eight) features to identify figures. While the emotion potential/EP played a top role for all seven figures, conscientiousness, and agreeableness were important to identify “Harry,” but not for example “Hermione.”

**Table 2:** Individual feature importances –as estimated by the Neural Net model– for the seven main figures.

**Figure/Feature**	**O**	**C**	**E**	**A**	**N**	**val**	**aro**	**EP**
Dobby	0.1	0.7	0.8	0.8	0.6	0.1	0.6	0.9
Dumbledore	0.7	0.4	0.4	0.7	0.8	0.1	0.9	0.8
Harry	0.1	0.8	0.4	0.7	0.1	0.1	0.7	0.9
Hagrid	0.7	0.1	0.4	0.6	0.6	0.1	0.9	0.7
Hermione	0.6	0.1	0.6	0.1	0.8	0.1	0.8	0.8
Snape	0.1	0.2	0.4	0.1	0.3	0.1	0.9	0.6
Voldemort	0.1	0.1	0.7	0.7	0.8	0.8	0.3	0.9

#### Figure Classification

Since the neural net model's excellent performance was obtained for the entire data set, a cross-validation not being possible given that each figure represents its own class, I ran a 2nd classification experiment. Lacking empirical data from human raters, in that experiment I used the “goodness/badness” of character for the 100 figures as a superordinate class label, evaluated on the basis of “Harry Potter” homepages that categorized them—as clearly as possible—as either “good” (e.g., “friend of Harry,” “the Weasleys”) or “bad” (e.g., “enemy of Harry,” “death eaters”). Thus, I could test the predictions from the emotional figure profile and pseudo-big5 computations against this empirical data^7^.

The data in Table 3a show a classification accuracy of just over 80% (for AdaBoost and kNN). This is not excellent, as for figure identification, but pretty good given the likely noisiness of the internet data and the complete novelty of the tool (i.e., 1st issue of “SentiArt” without any revisions yet). The rank scores for the seven predictors in Table 3b are interesting because they suggest features that played a major or minor role in this multivariate classification and point to potential weaknesses of the computational model. According to both the Information Gain Ratio and χ^2^ scores (both implemented in *Orange*) arousal and extraversion were vital predictors, followed by Neuroticism/Emotional Instability and Openness/Intellect. The other three predictors played only minor roles here. Thus, one feature from the emotional figure profile (arousal) and one from the “pseudo-big5” figure personality profile (extraversion) stand out in this exploratory binary classification. Basically, figures with high arousal (and neuroticism) scores have a high likelihood of being “bad,” while figures with a high extraversion (emotion potential and openness) score tend to be “good” characters. The features agreeableness, conscientiousness and valence did not help much in the present classification. Fine tuning of the VSM (e.g., increasing dimensionality) and/or label lists [e.g., using different labels or only labels that have a maximum “confidence”; cf. Turney and Littman's (2003)] may improve their classification strength, as might chosing another sample of figures from “Harry Potter” (e.g., only those that occur with a certain frequency). Before carrying out such fine-tuning studies, however, collecting empirical data is a priority from the neurocognitive poetics perspective.

**Table 3:** Results of the binary figure classification (“good” vs. “bad”); **(A)** F1 Scores for seven classifiers (stratified 10-fold cross-validation) with eight predictors (100 figures); **(B)** Rank scores of the importance of each of eight features.

**Method**		**F1**
AdaBoost		0.818%
kNN		0.815%
Random Forest		0.727%
Neural Network		0.713%
Naive Bayes		0.636%
SVM		0.545%
Logistic Regression		0.168%
**Feature/Importance rank**	**Information gain ratio**	***χ***^2^
Arousal	0.501	8.268
Extraversion	0.375	4.602
Neuroticism	0.157	1.875
Emotion potential	0.123	1.3
Openness	0.123	0.180
Agreeableness	0.031	0.018
Conscientiousness	0.031	0.018
Valence	0.031	0.018

## Discussion

In sum, applying the empirically validated techniques developed for SA of texts (study 1) to fiction characters (study 2) produced some interesting results with plausible face validity, high-accuracy identification of 100 figures and a decent classification accuracy regarding “goodness” of character data for those figures sampled from the internet. The emotional figure profiles and figure personality profiles of seven main characters from Harry Potter appear to have sufficient face validity to justify future empirical studies and cross-validation by experts. If replicated with other texts and figures this advanced SA opens numerous possibilities for research in digital literary studies, neurocognitive poetics, applied reading research, and other fields.

For example, a major issue in neurocognitive poetics is the investigation of the *immersion potential* of texts and other media (Lüdtke et al., 2014; Schlochtermeier et al., 2015) which correlates with a number of factors among which sympathy for and identification with protagonists seems central (Jacobs and Lüdtke, 2017). The emotions that readers experience during narrative comprehension depend upon psychological processes, such as identification with a protagonist and sympathy for story characters (e.g., Ferstl et al., 2005; Oatley, 2016; Jacobs and Willems, 2018). The likeability of stories depends on this, as Jose and Brewer (1984) already showed for children readers: the overall liking of a story indeed increased with greater identification, greater suspense, and greater liking of outcome. While young children (7 years) preferred positive outcomes regardless of the valence of the main character, older children (10–12) liked “happy endings” for good characters and negative endings for bad characters. To what extent the hybrid hero potential also contributes to this is a fascinating open empirical question that can now be investigated on the basis of predictions derived from emotional figure profiles and/or figure personality profiles.

Using *SentiArt* one can easily quantify a text's hypothetical immersion potential or the theoretical likeability of fiction characters, thus predict the outcome of experiments with human readers and test key hypotheses of the Neurocognitive Poetics Model of literary reading (Jacobs, 2015a,b), such as the fiction feeling hypothesis. It states that narratives with emotional contents invite readers more to be empathic with the protagonists and immerse in the text world (e.g., by engaging the affective empathy network of the brain), than do stories with neutral contents. In an fMRI study Hsu et al. (2014) tested and found support for this. Comparing the neural correlates of *post hoc* immersion ratings for fear-inducing vs. neutral passages from the *Harry Potter* series revealed that activity in the midcingulate cortex correlated more strongly with the ratings for the emotional than for the neutral passages. Descriptions of protagonists' pain or personal distress featured in the fear-inducing passages may have recruited the core structure of pain and affective empathy the more readers immersed in the text. Via *SentiArt* both the emotion potential of a key passage of text and the likeability of the character appearing in that passage (emotional figure profile, figure personality profile) can be computed and used for deriving testable predictions. Adequately combined with a scientific assessment of readers' personality profiles or emotional states (e.g., Calvo and Castillo, 2001) it can be used to predict not only emotional responses to narratives but also reading comprehension. The latter can indeed be facilitated when there is a felt match between readers and fictive characters, e.g., when highly extraverted participants read stories about, and rated the emotional experiences of, extraverted protagonists, with personalities similar to their own (Komeda et al., 2009).

## Conclusion, Limitations, and Outlook

A first general “take home message” from the present computational studies is that Turney and Littman's (2003) unsupervised learning approach termed “semantic orientation from association” still is a useful tool for VSM-based sentiment analyses of literary texts when access to published word lists providing valence ratings is difficult or impossible, or when these word lists do not adequately match the vocabulary of the text. *SentiArt*'s encouraging performance for this text material in Study 1 is promising: if replicated with other materials in future studies, it would mean that texts for which sophisticated English word-list based SATs like VADER are suboptimal (such as Shakespeare sonnets; cf. Jacobs, 2018b) can still undergo decent sentiment analyses. The condition is that one applies *SentiArt* in combination with an appropriate training corpus and VSM such as the present “wiki.en.vec” or—for “higher” literary English texts—the above mentioned GLEC (Jacobs, 2018a). This corpus comprises ~3.000 English texts (~650.000 types) spanning a range of genres from both fiction (prose & poetry) and non-fiction written by more than 130 authors (e.g., Darwin, Dickens, Shakespeare). A VSM based on this corpus was recently applied to predict the theoretically most beautiful line and the total emotion potential of Shakespeare sonnets (Jacobs, 2018b), or poems from Joyce and Eliot (Jacobs, 2018a). Since *SentiArt* also functions for other languages than English, for example German (Jacobs, 2017), and theoretically for any language for which training corpora and semantic vectors—but assumedly no appropriate word lists—are available, its application potential is vast. Of course, future computational and empirical studies must test its generalizability, validity, and reliability in other contexts.

In the light of previous results (e.g., Bestgen, 1994) the present results further suggest that an easy-to-compute lexical text feature (valence) can very well-predict a complex human performance such as when readers rate whether a text (segment) is “fearful,” “happy,” or “neutral.” In doing so they rely—implicitly or explicitly—on a great number of interacting lower- and higher-level text features (Jacobs, 2015a, 2018b; Jacobs et al., 2016a) that cannot all easily be computed (Jacobs, 2018b), but—at least in some contexts—approximated well-enough.

A second take home message more specifically concerns the fields of digital literary, distance and applied reading or neurocognitive poetics studies: application of a simple, easy-to-use VSM-based SAT produces promising results for predicting the hypothetical identification of readers with fiction characters and resulting emotional responses as well as reading comprehension. Computing emotional figure profiles and figure personality profiles for different figures and/or narratives—in combination with other quantitative narrative analyses of e.g., text cohesion, syntactic complexity or aesthetic potential (Jacobs, 2018b)—could thus help to better understand which paragraph or figure is most likely to drive emotional responses and facilitate comprehension.

An obvious limitation of the present computational studies—apart from having applied *SentiArt* to only 120 text samples from a single English book series (Study 1) and only one training corpus (Study 2)—is that it does not compute contextual polarity, i.e., it uses no “modifies polarity” or phrase-level features like negation. It also uses no lexical disambiguation method concerning words that can have several polarities. The excellent performance of *SentiArt* in Study 1 and the promising results from Study 2—at least for the present materials—suggest though that when both the training corpus and label lists are well-chosen, a simplistic, easy-to-use unsupervised SAT can do very well without additional computationally more costful analyses such as parsing, sense disambiguation or aspect-based SA (which all are still under dynamic development). This is especially interesting for researchers who have no substantial training in NLP methods but access to *fasttext* (Bojanowski et al., 2017) and large, representative training corpora (like about anybody these days; cf. Footnote 2).

Naturally, the present results must be replicated with other text materials and empirically verified before any general conclusions can be drawn. To what extent the training corpora, VSMs and label sets used by *SentiArt* also work for other literary texts (in other languages) is a fascinating issue for future studies. Finally, it is important to note that the present computational analyses are exploratory and can be used as a computational “*null model”* of the “sentiment” of verbal materials or the “personality” of fiction figures against which more sophisticated or general future models can be tested.

## Author Contributions

The author confirms being the sole contributor of this work and has approved it for publication.

## Conflict of Interest Statement

The author declares that the research was conducted in the absence of any commercial or financial relationships that could be construed as a potential conflict of interest.

## Footnotes

^1^The compound score is a normalized weighted composite score ranging from−1/negative to +1/positive. According to the authors, a word or text segment has a positive sentiment if its compound score > = 0.05, a negative one if it < = 0.05. If the score falls in between these two threshold values, the text is considered neutral.

^2^The easy-to-use procedure was tested during the workshop Plotting Poetry: Bringing Deep Learning to Computational Poetry Analysis‘(FU Berlin, sept 12-14th, 2018) where I trained 30 students from literature studies in SA methods.

^3^F1 is a weighted harmonic mean of precision and recall. Precision is the proportion of true positives among instances classified as positive. Recall is the proportion of true positives among all positive instances in the data.

^4^The model parameters were as follows: three hidden units, TanH activation function, learning rate of 0.1, no boosting, 10 iterations with a squared penalty function (cf. Jacobs and Lüdtke, 2017).

^5^Entropy R^2^ compares the log-likelihoods from the fitted model and a constant probability model: R^2^ = 1 – loglikelihood(model)/loglikelihood(0).

^6^Not all items used in the present pseudo-big5 model are shown here for better visibility (the original version used eight negative and six positive labels; see Appendix A in the Supplementary Material). Note that the ,‘negative' and ,‘positive' labels are not necessarily antonyms.

^7^For example, https://www.hp-lexicon.org/character/; https://www.ign.com/articles/2018/11/24/top-25-harry-potter-characters; https://www.pottermore.com/explore-the-story/mundungus-fletcher; https://www.hp-fc.de/hpfc/inhalte/de/personen/

## References

[B1] Altmann U. Bohrn I. C. Lubrich O. Menninghaus W. Jacobs A. M. (2012). The power of emotional valence - from cognitive to affective processes in reading. Front. Hum. Neurosci. 6:192. 10.3389/fnhum.2012.00192 22754519 PMC3385211

[B2] Altmann U. Bohrn I. C. Lubrich O. Menninghaus W. Jacobs A. M. (2014). Fact vs fiction—how paratextual information shapes our reading processes. Soc. Cogn. Affect. Neurosci. 9, 22–29. 10.1093/scan/nss098 22956671 PMC3871725

[B3] Andrews M. Vigliocco G. Vinson D. (2009). Integrating experien- tial and distributional data to learn semantic representations. Psychol. Rev. 116, 463–498. 10.1037/a0016261 19618982

[B4] Bestgen Y. (1994). Can emotional valence in stories be determined from words? Cogn. Emot. 8, 21–36. 10.1080/02699939408408926

[B5] Bestgen Y. Vincze N. (2012). Checking and boot-strapping lexical norms by means of word similarity indexes. Behav. Res. Methods 44, 998–1006. 10.3758/s13428-012-0195-z 22396137

[B6] Bird S. Klein E. Loper E. (2009). Natural Language Processing With Python. California, CA: O'Reilly Media.

[B7] Bojanowski P. Grave E. Joulin A. Mikolov T. (2017). Enriching word vectors with subword information. Trans. Assoc. Comput. Linguist. 5:51 10.1162/tacl_a_00051

[B8] Briesemeister B. B. Kuchinke L. Jacobs A. M. (2011). Discrete emotion norms for nouns: Berlin a ective word list (DENN – BAWL). Behav. Res. Methods 43, 441–448. 10.3758/s13428-011-0059-y 21416309

[B9] Calvo M. G. Castillo M. D. (2001). Bias in predictive inferences during reading. Discourse Process. 32, 43–71. 10.1207/S15326950DP3201_03

[B10] Conrad M. Recio G. Jacobs A. M. (2011). The time course of emotion effects in first and second language processing: across cultural ERP study with German–Spanish bilinguals. Front. Psychol. 2:351. 10.3389/fpsyg.2011.00351 22164150 PMC3230907

[B11] Costa P. T. Jr. McCrae R. R. (1988). From catalog to classification: Murray's needs and the five-factor model. J. Personal. Soc. Psychol. 55, 258–265. 10.1037/0022-3514.55.2.258

[B12] Deerwester S. Dumais S. T. Furnas G. W. Landauer T. K. Harshman R. (1990). Indexing by latent semantic analysis. J. Am. Soc. Inform. Sci. 41, 391–407. 10.1002/(SICI)1097-4571(199009)41:6<391::AID-ASI1>3.0.CO;2-9

[B13] Demsar J. Curk T. Erjavec A. Gorup C. Hocevar T. Milutinovic M. (2013). Orange: data mining toolbox in python. J. Mach. Learn. Res. 14, 2349–2353.

[B14] Egloff M. Picca D. Curran K. (2016). How IBM watson can help us understand character in Shakespeare: a cognitive computing approach to the plays, in Digital Humanities 2016: Conference Abstracts. Jagiellonian University and Pedagogical University, Kraków, 488–492.

[B15] Ekman P. (1999). Basic emotions, in Handbook of Cognition and Emotion, eds Dalgleish T. Power M. (Chichester: John Wiley and Sons), 45–60. 10.1002/0470013494.ch3

[B16] Elsner M. (2012). Character-based kernels for nov- elistic plot structure, in Proceedings of the 13th Conference of the European Chapter of the Association for Computational Linguistics, EACL'12, Stroudsburg, PA Association for Computational Linguistics, 634–644,.

[B17] Ferrucci D. A. (2012). Introduction to ‘This is Watson'. IBM J. Res. Dev. 56:1 10.1147/JRD.2012.2184356

[B18] Ferstl E. C. Rinck M. von Cramon D. Y. (2005). Emotional and temporal aspects of situa- tion model processing during text comprehension: an event-related fMRI study. J. Cogn. Neurosci. 17, 724–739. 10.1162/0898929053747658 15904540

[B19] Ganascia J.-G. (2015). The logic of the big data turn in digital literary studies. Front. Digital Human. 2:7 10.3389/fdigh.2015.00007

[B20] Goldberg L. R. (1992). The development of markers of the Big-Five factor structure. Psychol. Assess. 4, 26–42. 10.1037/1040-3590.4.1.26

[B21] Harris Z. S. (1951). Methods in Structural Linguistics. Chicago: University of Chicago Press. Available online at: http://archive.org/details/structurallingui00harr

[B22] Hofmann M. J. Tamm S. Braun M. M. Dambacher M. Hahne A. Jacobs A. M. (2008). Conflict monitoring engages the mediofrontal cortex during nonword processing. Neuroreport 19, 25–29. 10.1097/WNR.0b013e3282f3b134 18281887

[B23] Hollis G. Westbury C. Lefsrud L. (2017). Extrapolating human judgments from skip-gram vector representations of word meaning. Quart. J. Exp. Psychol. 2017, 1–45. 10.1080/17470218.2016.1195417 27251936

[B24] Hsu C.-T. Jacobs A. M. Altmann U. Conrad M. (2015c). The magical activation of left amygdala when reading Harry Potter: an fMRI study on how descriptions of supra-natural events entertain and enchant. PLoS ONE 10:e0118179. 10.1371/journal.pone.0118179 25671315 PMC4324997

[B25] Hsu C.-T. Jacobs A. M. Conrad M. (2015b). Can Harry Potter still put a spell on us in a second language? An fMRI study on reading emotion-laden literature in late bilinguals. Cortex 63, 282–295. 10.1016/j.cortex.2014.09.002 25305809

[B26] Hsu C. T. Conrad M. Jacobs A. M. (2014). Fiction feelings in Harry Potter: Haemodynamic response in the mid-cingulate cortex correlates with immersive reading experience. NeuroReport 25, 1356–1361. 10.1097/WNR.0000000000000272 25304498

[B27] Hsu C. T. Jacobs A. M. Citron F. Conrad M. (2015a). The emotion potential of words and passages in reading Harry Potter - An fMRI study. Brain Lang. 142, 96–114. 10.1016/j.bandl.2015.01.011 25681681

[B28] Hu M. Liu B. (2004). Mining and summarizing customer reviews, in Proceedings of the Tenth ACM SIGKDD International Conference on Knowledge Discovery and Data Mining, eds Kim W. Kohavi R. (Washington, DC: ACM Press), 168–177. 10.1145/1014052.1014073

[B29] Hutto C. J. Gilbert E. E. (2014). VADER: a parsimonious rule-based model for sentiment analysis of social media rext, in Eighth International Conference on Weblogs and Social Media (ICWSM-14), Ann Arbor, MI.

[B30] Jacobs A. (2015c). The scientific study of literary experience: sampling the state of the art. Sci. Study Lit. 5, 139–170. 10.1075/ssol.5.2.01jac

[B31] Jacobs A. M. (2011). Neurokognitive Poetik: elemente eines modells des literarischen lesens (Neurocognitive poetics: Elements of a model of literary reading), in Gehirn und Gedicht: Wie wir unsere Wirklichkeiten konstruieren (Brain and Poetry: How We Construct Our Realities), eds Schrott R. Jacobs A. M. (München: Carl Hanser Verlag, 492–520.

[B32] Jacobs A. M. (2015a). Towards a neurocognitive poetics model of literary reading, in Towards a Cognitive Neuroscience of Natural Language Use, ed Willems R. (Cambridge: Cambridge University Press), 135–159. 10.1017/CBO9781107323667.007

[B33] Jacobs A. M. (2015b). Neurocognitive poetics: methods and models for investigating the neuronal and cognitive-affective bases of literature reception. Front. Hum. Neurosci. 9:186. 10.3389/fnhum.2015.00186 25932010 PMC4399337

[B34] Jacobs A. M. (2016). The scientific study of literary experience and neuro-behavioral responses to literature: reply to commentaries. Sci. Study Lit. 6, 164–174. 10.1075/ssol.6.1.08jac

[B35] Jacobs A. M. (2017). Quantifying the beauty of words: a neurocognitive poetics perspective. Front. Hum. Neurosci. 11:622. 10.3389/fnhum.2017.00622 29311877 PMC5742167

[B36] Jacobs A. M. (2018a). The gutenberg english poetry corpus: exemplary quantitative narrative analyses. Front. Digit. Humanit. 5:5 10.3389/fdigh.2018.00005

[B37] Jacobs A. M. (2018b). (Neuro-)cognitive poetics and computational stylistics. Sci. Study Lit. 8:1, 164–207. 10.1075/ssol.18002.jac

[B38] Jacobs A. M. Hofmann M. J. Kinder A. (2016a). On elementary affective decisions: to like or not to like, that is the question. Front. Psychol. 7:1836 10.3389/fpsyg.2016.01836 27933013 PMC5122311

[B39] Jacobs A. M. Kinder A. (2017). The brain is the prisoner of thought: A machine-learning assisted quantitative narrative analysis of literary metaphors for use in Neurocognitive Poetics. Metaphor Symbol 32, 139–160. 10.1080/10926488.2017.1338015

[B40] Jacobs A. M. Kinder A. (2018). What makes a metaphor literary? Answers from two computational studies. Metaphor Symbol 33, 85–100. 10.1080/10926488.2018.1434943

[B41] Jacobs A. M. Lüdtke J. (2017). Immersion into narrative and poetic worlds: a neurocognitive poetics perspective, in Handbook of Narrative Absorption, eds Kuijpers M. Hakemulder F. (Amsterdam: John Benjamins), 69–96. 10.1075/lal.27.05jac

[B42] Jacobs A. M. Lüdtke J. Aryani A. Meyer-Sickendiek B. Conrad M. (2016b). Mood- empathic and aesthetic responses in poetry reception: a model-guided, multilevel, multimethod approach. Sci. Study Lit. 6, 87–130. 10.1075/ssol.6.1.06jac

[B43] Jacobs A. M. Schuster S. Xue S. Lüdtke J. (2017). What's in the brain that ink may character: a quantitative narrative analysis of Shakespeare's 154 sonnets for use in neurocognitive poetics. Sci. Study Lit. 7, 4–51. 10.1075/ssol.7.1.02jac

[B44] Jacobs A. M. Võ M. L.-H. Briesemeister B. B. Conrad M. Hofmann M. J. Kuchinke L.. (2015). 10 years of BAWLing into affective and aesthetic processes in reading: what are the echoes? Front. Psychol. 6:714. 10.3389/fpsyg.2015.00714 26089808 PMC4452804

[B45] Jacobs A. M. Willems R. (2018). The fictive brain: neurocognitive correlates of engagement in literature. Rev. General Psychol. 22, 147–160. 10.1037/gpr0000106

[B46] Jose P. E. Brewer W. F. (1984). Development of story liking: character identification, suspense, and outcome resolution. Dev. Psychol. 20, 911–924. 10.1037/0012-1649.20.5.911

[B47] Klinger R. (2018). Digitale Modellierung von Figurenkomplexität am Beispiel des Parzival von Wolfram von Eschenbach. Available online at: http://www.ims.uni-stuttgart.de/institut/mitarbeiter/klingern

[B48] Komeda H. Kawasaki M. Tsunemi K. Kusumi T. (2009). Differences between estimating protagonists' emotions and evaluating readers' emotions in narrative comprehension. Cogn. Emot. 23, 135–151. 10.1080/02699930801949116

[B49] Liu B. (2015). Sentiment Analysis: Mining Opinions, Sentiments, and Emotions. Cambridge: Cambridge University Press 10.1017/CBO9781139084789

[B50] Lüdtke J. Meyer-Sickendiek B. Jacobs A. M. (2014). Immersing in the stillness of an early morning: testing the mood empathy hypothesis in poems. Psychol. Aesthetics Creativity Arts, 8, 363–377. 10.1037/a0036826

[B51] Mandera P. Keuleers E. Brysbaert M. (2015). How useful are corpus-based methods for extrapolating psycholinguistic variables? Quart. J. Exp. Psychol. 68, 1623–1642. 10.1080/17470218.2014.988735 25695623

[B52] Mikolov T. Chen K. Corrado G. Dean J. (2013). Efficient Estimation of Word Representations in Vector Space. Retrieved from https://arxiv.org/abs/1301.3781

[B53] Miller G. A. (1995). WordNet: a lexical database for English. Commun. ACM 38, 39–41. 10.1145/219717.219748

[B54] Nalisnick E. Baird H. (2013). Character-to-character sentiment analysis in Shakespeare's plays, in Proceedings of the 51st Annual Meeting of the Association for Computational Linguistics (Sofia), 479–483.

[B55] Nicklas P. Jacobs A. M. (2017). Rhetorics, neurocognitive poetics and the aesthetics of adaptation. Poetics Today 38, 393–412. 10.1215/03335372-3869311

[B56] Norman W. T. (1963). Toward an adequate taxonomy of personality attributes: replicated factor structure in peer nomination personality ratings. J. Abnorm. Soc. Psychol. 66, 574–583. 10.1037/h0040291 13938947

[B57] Oatley K. (2016). Fiction: simulation of social worlds. Trends Cogn. Sci. 20, 618–628. 10.1016/j.tics.2016.06.002 27449184

[B58] Osgood C. E. Suci G. J. Tannenbaum P. H. (1957). The Measurement of Meaning. Urbana, IL: University of Illinois Press.

[B59] Recchia G. Louwerse M. M. (2015). Reproducing affective norms with lexical co-occurrence statistics: predicting valence, arousal, and dominance. Quart. J. Exp. Psychol. 68, 1584–1598. 10.1080/17470218.2014.941296 24998307

[B60] Rowling J. K. (1997). Harry Potter and the Philosopher's Stone. London: Bloomsbury.

[B61] Rowling J. K. (1998). Harry Potter and the Chamber of Secrets. London: Bloomsbury.

[B62] Rowling J. K. (1999). Harry Potter and the Prisoner of Azkaban. London: Bloomsbury.

[B63] Rowling J. K. (2000). Harry Potter and the Goblet of Fire. London: Bloomsbury.

[B64] Rowling J. K. (2003). Harry Potter and the Order of the Phoenix. London: Bloomsbury.

[B65] Rowling J. K. (2005). Harry Potter and the Half Blood Prince. London: Bloomsbury.

[B66] Rowling J. K. (2007). Harry Potter and the Deathly Hallows. London: Bloomsbury.

[B67] Schlochtermeier L. H. Pehrs C. Kuchinke L. Kappelhoff H. Jacobs A. M. (2015). Emotion processing in different media types: realism, complexity, and immersion. J. Syst. Integr. Neurosci. 1, 41–47. 10.15761/JSIN.1000109

[B68] Schmidtke D. S. Schröder T. Jacobs A. M. Conrad M. (2014). ANGST: affective norms for German sentiment terms, derived from the affective norms for English words. Behav. Res. Methods 46, 1108–1118. 10.3758/s13428-013-0426-y 24415407

[B69] Schroeder S. Würzner K. M. Heister J. Geyken A. Kliegl R. (2015). childLex: a lexical database of German read by children. Behav. Res. Methods 47, 1085–1094. 10.3758/s13428-014-0528-1 25319039

[B70] Schrott R. Jacobs A. M. (2011). Gehirn und Gedicht: Wie wir unsere Wirklichkeiten konstruieren (Brain and Poetry: How We Construct Our Realities). München: Hanser.

[B71] Simonton D. K. (1989). Shakespeare's Sonnets: a case of and for single–case historiometry. J. Personal. 57, 695–721. 10.1111/j.1467-6494.1989.tb00568.x

[B72] Stone P. J. Dunphy D. C. Smith M. S. Ogilvie D. M. (1966). The General Inquirer: A Computer Approach to Content Analysis. Cambridge: Massachusetts Institute of Technology Press.

[B73] Taboada M. Brooke J. Tofiloski M. Voll K. Stede M. (2011). Lexicon-based methods for sentiment analysis. Comput. Linguist. 37, 267–307. 10.1162/COLI_a_00049

[B74] Thompson E. R. (2008). Development and validation of an international English Big-Five Mini-Markers. Personal. Individ. Differ. 45, 542–548. 10.1016/j.paid.2008.06.013

[B75] Turney P. D. (2001). Mining the Web for synonyms: PMI-IR versus LSA on TOEFL, in Proceedings of the 12th European Conference on Machine Learning. Berlin: Springer-Verlag, 491–502. 10.1007/3-540-44795-4_42

[B76] Turney P. D. Littman M. L. (2003). Measuring praise and criticism: inference of semantic orientation from association. ACM Trans. Inform. Syst. 21, 315–346. 10.1145/944012.944013

[B77] Veltkamp G. M. Recio G. Jacobs A. M. Conrad M. (2012). Is personality modulated by language? Int. J. Bilingual. 17, 496–504. 10.1177/1367006912438894

[B78] Võ M. L. H. Conrad M. Kuchinke L. Hartfeld K. Hofmann M. J. Jacobs A. M. (2009). The berlin affective word list reloaded (BAWL-R). Behav. Res. Methods 41, 534–539. 10.3758/BRM.41.2.534 19363195

[B79] Võ M. L. H. Jacobs A. M. Conrad M. (2006). Cross-validating the Berlin affective word list. Behav. Res. Methods 38, 606–609. 10.3758/BF03193892 17393831

[B80] Warriner A. B. Kuperman V. Brysbaert M. (2013). Norms of valence, arousal, and dominance for 13,915 English lemmas. Behav. Res. Methods 45, 1191–1207. 10.3758/s13428-012-0314-x 23404613

[B81] Westbury C. (2016). Pay no attention to that man behind the curtain. Mental Lexicon 11, 350–374. 10.1075/ml.11.3.02wes

[B82] Westbury C. Keith J. Briesemeister B. B. Hofmann M. J. Jacobs A. M. (2015). Avoid violence, rioting, and outrage; approach celebration, delight, and strength: using large text corpora to compute valence, arousal, and the basic emotions. Quart. J. Exp. Psychol. 68, 1599–1622. 10.1080/17470218.2014.970204 26147614

[B83] Westbury C. F. Shaoul C. Hollis G. Smithson L. Briesemeister B. B. Hofmann M. J.. (2013). Now you see it, now you don't: on emotion, context, and the algorithmic prediction of human imageability judgments. Front. Psychol. 4:991. 10.3389/fpsyg.2013.00991 24421777 PMC3872786

[B84] Whissell C. Fournier M. Pelland R. Weir D. Makarec K. (1986). A dictionary of affect in language: IV. Reliability, validity, and applications. Percept. Motor Skills 62, 875–888. 10.2466/pms.1986.62.3.875

[B85] Wiebe J. Wilson T. Cardie C. (2005). Annotating expressions of opinions and emotions in language. Lang. Resourc. Eval. 39, 165–210. 10.1007/s10579-005-7880-9

[B86] Willems R. Jacobs A. M. (2016). Caring about Dostoyevsky: the untapped potential of studying literature. Trends Cogn. Sci. 20, 243–245. 10.1016/j.tics.2015.12.009 26809726

[B87] Wundt W. M. (1874). Grundzüge der Physiologischen Psychologie. Leipzig: Engelmann.

