# Supplementary material for: Sentiment Analysis for Words and Fiction Characters From the Perspective of Computational (Neuro-)Poetics
Source: Front Robot AI. 2019 Jul 17;6:53. doi: 10.3389/frobt.2019.00053 (PMC7805775; doi:10.3389/frobt.2019.00053)
Supplement: Supplementary file 1 [file Data_Sheet_1.docx]

**Appendix**

**A. Six positive and eight negative labels for computing the ‘Agreeableness’ values**

['beschwichtigend'/CONCILIATORY, ’freundlich'/FRIENDLY, 'mitfühlend'/CARING, 'liebevoll'/AFFECTIONATE, 'warm'/WARM, ’kumpelhaft’/CHUMMY]

['ablehnend'/HOSTILE, 'argwöhnisch'/DISTRUSTFUL, 'arrogant'/ARROGANT, 'gemein'/MEAN, 'gierig'/GREEDY, 'gefühllos'/DEADHEARTED, 'trotzig'/DEFIANT, 'verdrießlich'/GRUMPY]

**B. Sample of 100 Figures from Harry Potter with ‚good/bad‘ labels based on an internet search**

As an example, on <https://www.ign.com/articles/2019/03/28/top-25-harry-potter-characters> (june 4th, 2019) ‘Dobby’ is described as ‘In the books, we saw more of how kind-hearted and loyal Dobby was, particularly towards Harry Potter, to whom he was indebted for his freedom. But even those who only knew Dobby from The Chamber of Secrets film were probably affected by his sacrifice in The Deathly Hallows Part 1, where he proved crucial in freeing Harry and his friends from a horrific scenario, only to fall victim to Bellatrix Lestrange in the process. The fact that Dobby, in his dying moments, clearly had no regrets about literally giving his all for his friend Harry resulted in one of the most touching moments in the series.’ On the basis of this description and others from the homepages mentioned in Footnote 6 above, the author categorized ‘Dobby’ as good. It should be noted that the binary categorization done by the author on the basis of judgments/descriptions by users of the homepages as well as 'experts', which is based on a large consensus of readers and viewers of the series, is a non-optimal pragmatic and tentative procedure which could be cross-validated by an empirical study in the future.

| **Figure** | **good/bad** |
| --- | --- |
| Arthur | g |
| Alecto | b |
| Amycus | b |
| Augustus | b |
| Avery | b |
| Barny | g |
| Bartemius | b |
| Bellatrix | b |
| Bill | g |
| Cedric | g |
| Charlie | g |
| Colin | g |
| Crabbe | b |
| Dean | g |
| Demelza | g |
| Dennis | g |
| Dobby | g |
| Dolohow | b |
| Dolores | b |
| Dragomir | b |
| Dudley | g |
| Dumbledore | g |
| Eileen | b |
| Ernie | g |
| Filch | b |
| Finnigan | g |
| Fleur | g |
| Flitwick | g |
| Figg | g |
| Fred | g |
| Fridwulfa | b |
| Fudge | b |
| George | g |
| Gibbon | b |
| Ginny | g |
| Gornuk | b |
| Goyle | b |
| Gregory | b |
| Greyback | b |
| Grindelwald | b |
| Harry | g |
| Hagrid | g |
| Hermine | g |
| Higgs | b |
| Hooch | g |
| Hugo | g |
| Karkaroff | b |
| Kreacher | b |
| Krum | g |
| Lily | g |
| Lockhart | g |
| Loxias | b |
| Lucius | b |
| Luna | g |
| Lupin | g |
| Mafalda | g |
| Magda | b |
| Malfoy | b |
| Malkin | g |
| Mason | b |
| McGonagall | g |
| McLaggen | g |
| Michael | b |
| Millicent | b |
| Moody | g |
| Mulciber | b |
| Myrte | g |
| Neville | g |
| Nick | g |
| Nott | b |
| Nymphadora | b |
| Padma | g |
| Parvati | g |
| Percy | g |
| Pettigrew | b |
| Petunia | b |
| Pius | b |
| Pomfrey | g |
| Regulus | b |
| Rita | b |
| Rodolphus | b |
| Roger | g |
| Romilda | b |
| Ron | g |
| Ronan | g |
| Rosier | b |
| Rosmerta | g |
| Salazar | b |
| Scrimgeour | b |
| Seamus | g |
| Slughorn | g |
| Snape | g |
| Sprout | b |
| Stan | b |
| Susan | g |
| Vernon | b |
| Voldemort | b |
| Weasley | g |
| Wilbert | b |
| Wilkes | b |
